# Reading Frame Correction by Targeted Genome Editing Restores Dystrophin Expression in Cells From Duchenne Muscular Dystrophy Patients

**DOI:** 10.1038/mt.2013.111

**Published:** 2013-06-04

**Authors:** David G Ousterout, Pablo Perez-Pinera, Pratiksha I Thakore, Ami M Kabadi, Matthew T Brown, Xiaoxia Qin, Olivier Fedrigo, Vincent Mouly, Jacques P Tremblay, Charles A Gersbach

**Affiliations:** 1 Department of Biomedical Engineering, Duke University, Durham, North Carolina, USA; 2 Institute for Genome Sciences and Policy, Duke University, Durham, North Carolina, USA; 3 Thérapie des maladies du muscle strié/Institut de Myologie UM76, Université Pierre et Marie Curie, INSERM U974; CNRS UMR 7215, Paris, France; 4 Unité de Recherche en Génétique Humaine, Centre de Recherche de CHUL, CHUQ, Faculté de Médecine, Université Laval, Québec, Québec, Canada; 5 Department of Orthopaedic Surgery, Duke University Medical Center, Durham, North Carolina, USA

## Abstract

Genome editing with engineered nucleases has recently emerged as an approach to correct genetic mutations by enhancing homologous recombination with a DNA repair template. However, many genetic diseases, such as Duchenne muscular dystrophy (DMD), can be treated simply by correcting a disrupted reading frame. We show that genome editing with transcription activator-like effector nucleases (TALENs), without a repair template, can efficiently correct the reading frame and restore the expression of a functional dystrophin protein that is mutated in DMD. TALENs were engineered to mediate highly efficient gene editing at exon 51 of the dystrophin gene. This led to restoration of dystrophin protein expression in cells from Duchenne patients, including skeletal myoblasts and dermal fibroblasts that were reprogrammed to the myogenic lineage by MyoD. Finally, exome sequencing of cells with targeted modifications of the dystrophin locus showed no TALEN-mediated off-target changes to the protein-coding regions of the genome, as predicted by *in silico* target site analysis. This strategy integrates the rapid and robust assembly of active TALENs with an efficient gene-editing method for the correction of genetic diseases caused by mutations in non-essential coding regions that cause frameshifts or premature stop codons.

## Introduction

Genome editing with engineered site-specific endonucleases has emerged as a new technology to selectively replace or correct disrupted genes, in contrast to conventional gene therapy methods of gene addition.^1,2^ The recent development of transcription activator-like effector nucleases (TALENs) has dramatically advanced genome editing due to their high rate of successful and efficient genetic modification.^2,3,4,5,6,7,8,9,10,11,12,13^ TALENs are engineered fusion proteins of the catalytic domain of the endonuclease *Fok*I and a designed TALE DNA-binding domain that can be targeted to a custom DNA sequence.^5,6^ The TALE domain consists of an array of repeat variable diresidue modules, each of which specifically recognizes a single base pair of DNA.^3,4^ Repeat variable diresidue modules can be arranged in any order to assemble an array that recognizes a defined sequence.^3,4^ Site-specific double-strand breaks are created when two independent TALENs bind to adjacent DNA sequences, thereby permitting dimerization of *Fok*I and cleavage of the target DNA.^2^ This targeted double-strand break stimulates cellular DNA repair through either homology-directed repair (HDR) or the non-homologous end joining (NHEJ) pathway. HDR uses a donor DNA template to guide repair and can be used to create specific sequence changes to the genome, including the targeted addition of whole genes. In contrast, the template-independent re-ligation of DNA ends by NHEJ is a stochastic, error-prone repair process that introduces random microinsertions and microdeletions (indels) at the DNA breakpoint.

Strategies for the correction of human genes have been based primarily on HDR, the efficiency of which is dependent on cell-cycle state and delivery of an exogenous DNA template.^14,15,16,17,18^ In many cases, antibiotic selection is used in tandem with genome editing for gene correction in cell types with low levels of HDR repair.^15,16,17^ Although HDR is extremely valuable for restoring the complete coding sequence of the mutant gene, evidence from studies investigating oligonucleotide-mediated exon skipping and pharmacologic read-through of premature stop codons demonstrate that restoring expression of fully or partially functional truncated proteins can provide therapeutic benefit for many diseases.^19,20^ Therefore indels resulting from NHEJ-mediated gene repair could restore a disrupted reading frame or remove a premature stop codon and ameliorate the symptoms of these genetic diseases. This approach would result in permanent gene correction, in contrast to pharmacologic approaches that act transiently at the level of mRNA splicing or translation.

Thus far, NHEJ has been used in human cells to disrupt genes^21^ or to delete chromosomal segments,^22,23^ although it has been proposed that genetic mutations created by endonucleases could be used to restore an aberrant reading frame.^24,25^ In this study, we provide the first example of the restoration of protein expression from an endogenous mutated gene through template-free NHEJ-mediated DNA repair. Duchenne muscular dystrophy (DMD), the most common monogenic hereditary disease, is caused by defects in the gene encoding dystrophin. The majority of dystrophin mutations that cause DMD are deletions of exons that disrupt the reading frame.^20^ However, deletion of internal dystrophin exons that retain the proper reading frame causes the less severe Becker muscular dystrophy. This has led to efforts to restore the disrupted dystrophin reading frame in DMD patients by skipping non-essential exons during mRNA splicing, thereby producing internally deleted, but still partially or fully functional, dystrophin proteins.^19,26,27^ In contrast to a transient method targeting the dystrophin mRNA, the correction of the dystrophin reading frame in the genome by transiently expressed TALENs would lead to permanently restored dystrophin expression by each modified cell and all of its progeny. Notably, exon 51 is frequently adjacent to frame-disrupting deletions in DMD patients and has been targeted in clinical trials for oligonucleotide-based exon skipping with promising early therapeutic results.^19,26,27^ An ongoing clinical trial for the exon 51 skipping compound eteplirsen recently reported a significant functional benefit across 48 weeks, with an average of 47% dystrophin-positive fibers compared with baseline. Therefore, this class of DMD mutations is ideally suited for permanent correction by NHEJ-based genome editing.

This study investigates template-free gene correction by using TALENs to restore aberrant reading frames through the introduction of indels into exon 51 of the dystrophin gene. Accordingly, we designed and validated an optimized TALEN that targets exon 51. The optimized TALEN was transfected into human DMD cells and shown to mediate efficient gene modification and conversion to the correct reading frame. Furthermore, protein restoration was concomitant with frame restoration and could be detected in a bulk population of TALEN-treated cells. The high specificity of the optimized TALEN was demonstrated by *in silico* analysis, cytotoxicity assays, and exome sequencing of clonally derived modified cells.

## Results

### Design and validation of TALENs targeted to the dystrophin gene

**Figure 1:**
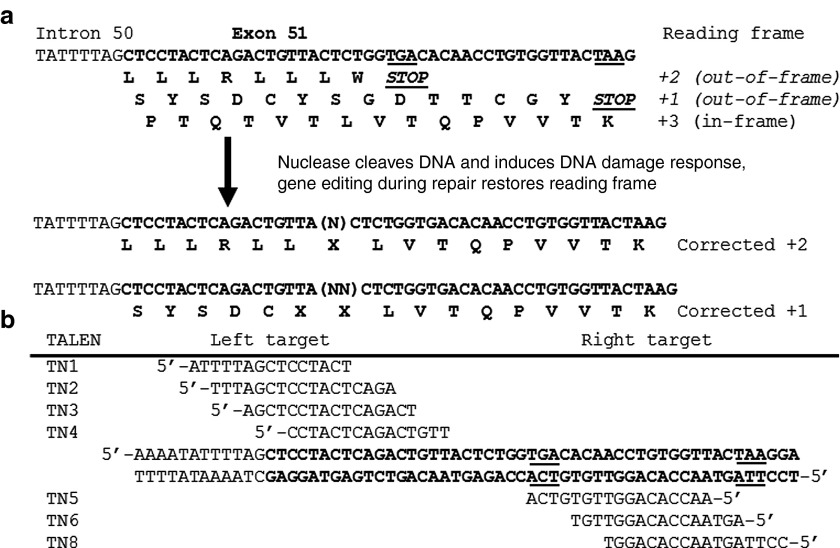
**Design of TALENs targeted to exon 51 of the human dystrophin gene**. (**a**) The possible reading frames of human dystrophin exon 51 and expected amino acid sequences after genome editing. (**b**) Combinations of TALEN pairs were designed to target immediately upstream of either out-of-frame stop codon (underlined) in exon 51 (bold) of the human dystrophin gene. TALEN, transcription activator-like effector nuclease.

**Figure 2:**
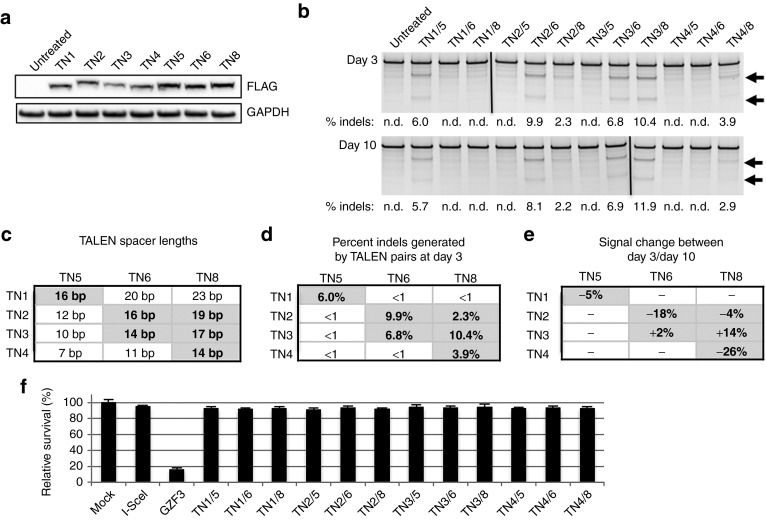
**Validation and characterization of TALENs**. (**a**) Each TALEN construct was transfected independently into HEK293T cells to confirm full-length expression. All TALENs were the expected size of ~95–110 kDa. (**b**) Combinations of TALENs were cotransfected into HEK293T cells to screen for highly active TALEN pairs. Gene modification frequency was monitored at day 3 and 10 to assess stable gene modification. Arrows denote expected cleavage band sizes indicative of NHEJ activity. (**c**) Summary of TALEN spacer lengths. (**d**) Measured gene modification rates detected by the Surveyor assay from day 3, data in **b**. (**e**) Measured indel signal changes between day 3 and 10 from the data in **b**. (**f**) Cytotoxicity assay in HEK293T cells for all TALEN combinations. I-SceI is a non-toxic meganuclease and GZF3 is a zinc finger nuclease known to be cytotoxic to human cells. GAPDH, glyceraldehyde 3-phosphate dehydrogenase; n.d., not detected; NHEJ, non-homologous end joining; TALEN, transcription activator-like effector nuclease.

To evaluate TALEN-mediated genetic correction by NHEJ, several TALENs were designed to target exon 51 in the dystrophin gene. TALEN target sites were chosen immediately upstream of the two possible out-of-frame stop codons (**Figure 1a**), such that insertions or deletions could restore the dystrophin reading frame in either disrupted frame. Variable lengths of spacers between TALEN monomers and TALEN repeat variable diresidue array lengths were tested to optimize nuclease activity (**Figure 1b**, **Supplementary Figure S1**), as done previously.^6^ Western blots confirmed full-length and robust expression of the TALENs following transfection of TALEN-encoding plasmids into HEK293T cells (**Figure 2a**). All combinations of left and right TALENs were transfected into HEK293T cells and the genomic DNA was assessed for modification by the Surveyor assay, which can detect the frequency of allelic modifications with a dynamic range of ~1–50%. Several TALENs with spacers of 14–19 bp were highly active with gene-editing efficiencies exceeding modification of 10% of total alleles (**Figure 2b**–**d**), consistent with previous observations.^6,7,10^ The gene-editing frequencies were stable from day 3 to day 10 (**Figure 2b**,**e**), confirming that these TALENs are well tolerated in human cells.^6,7,10^ Furthermore, the engineered TALENs showed minimal cytotoxicity in human cells similar to the well-characterized non-cytotoxic homing endonuclease I-SceI (**Figure 2f**, **Supplementary Figure S2**).^7,28^ TN3/8 was the most highly active and well-tolerated TALEN pair and therefore was used for subsequent experiments (**Supplementary Figure S3**).

### TN3/8 mediates high efficiency conversion to all three reading frames

**Figure 3:**
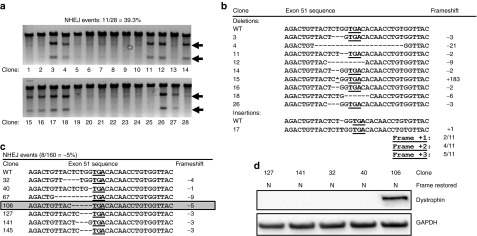
**Genetic correction of aberrant dystrophin reading frames by TALEN-mediated genome editing**. (**a**) Isogenic clones were derived from human skeletal myoblasts treated with 10 μg of each plasmid encoding TN3/8 and screened using the Surveyor assay to detect mutant alleles in reference to the parent (untreated) genomic DNA. Arrows denote expected cleavage band sizes indicative of NHEJ activity. (**b**) Sanger sequencing of the TALEN target site in exon 51 in mutant clones identified in **a**. (**c**) DMD human myoblast cell line 1 was treated with 10 μg of each plasmid encoding the TN3/8 TALEN pair and isogenic clones were subsequently derived. Sanger sequencing was used to identify clones with small insertion or deletion mutations at the exon 51 genomic locus characteristic of NHEJ. Clone 106 had a 5-bp deletion expected to restore the reading frame (boxed). All other clones had deletions that were not expected to result in corrective frameshift events. (**d**) Clonal cell populations with NHEJ events detected at exon 51 were cultured in differentiation conditions for 7 days and analyzed by western blot for dystrophin expression at the expected molecular weight (412 kDa). DMD, Duchenne muscular dystrophy; GAPDH, glyceraldehyde 3-phosphate dehydrogenase; NHEJ, non-homologous end joining; TALEN, transcription activator-like effector nuclease; WT, wild-type.

NHEJ-based gene modification is expected to create indels of random length and therefore should cause conversion to any of the three reading frames in an exonic sequence. To validate the overall gene modification rate and possible reading frames generated following TALEN-induced NHEJ, clonal cell populations were derived from human skeletal myoblasts that had been electroporated with TN3/8-encoding plasmids. These clones were assayed for NHEJ events occurring at the dystrophin exon 51 locus using the Surveyor assay to detect sequence differences relative to untreated cells (**Figure 3a**). Eleven of 28 (39%) clonal cell populations were modified and subsequent sequencing of the alleles from these clones confirmed indels characteristic of NHEJ (**Figure 3b**). Similar to other studies with TALENs, deletions were heavily favored.^29^ The random length of these indels verifies that conversion to any of the three reading frames is possible. The conversion rate to any one of the three reading frames was observed to be roughly proportional to the expected one-third of the total NHEJ events (**Figure 3b**). Interestingly, several small deletions were observed that did not alter the original reading frame, demonstrating that this approach could be used to delete aberrant stop codons (**Figure 3b**).

### Reading frame correction leads to restored protein expression

We next assessed whether correction of the dystrophin reading frame by TALEN-mediated NHEJ results in restored dystrophin protein expression. Immortalized human myoblasts derived from DMD patients with a frame-disrupted dystrophin gene caused by deletion of exons 48-50(Δ48-50) were electroporated with plasmids encoding TN3/8. Clonal cell populations were isolated and screened by PCR amplification of genomic DNA and Sanger sequencing to identify indels characteristic of NHEJ. In this experiment, ~5% of clones contained modifications in exon 51, including one clone with an NHEJ event expected to correct the dystrophin reading frame (**Figure 3c**, **Supplementary Figure S4**). Following myogenic differentiation, restored dystrophin protein expression was detected by western blot at its predicted size (~412 kDa) only in the corrected clone, and not in clones with non-corrective NHEJ events (**Figure 3d**). These data demonstrate that NHEJ events that restore the dystrophin reading frame also rescue dystrophin protein expression.

### TALEN-mediated genetic correction in bulk-treated DMD myoblasts

**Figure 4:**
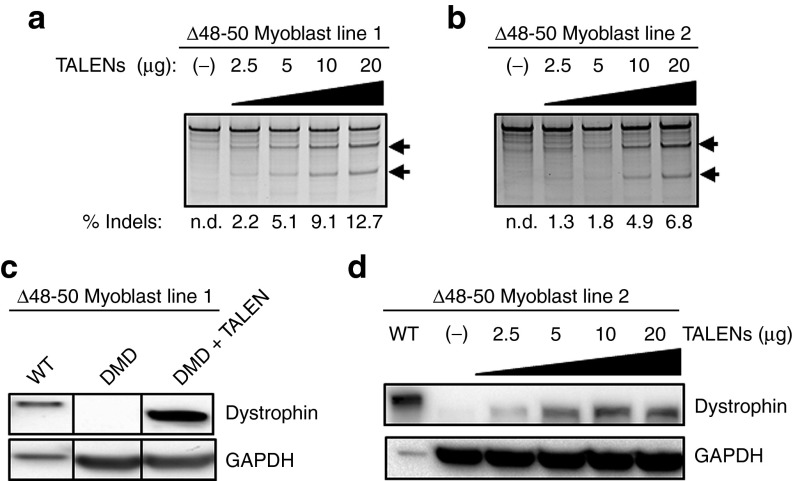
**Efficient genetic modification and protein restoration in a bulk population of cells treated with TN3/8**. (**a**,**b**) Dose-dependent response of NHEJ activity with increasing amounts of TALEN pair TN3/8 measured by the Surveyor assay after transfection of the indicated amount of each TALEN plasmid into two different DMD myoblast lines, each carrying a novel deletion of exons 48-50 (Δ48-50). Arrows denote expected cleavage band sizes indicative of NHEJ activity. (**c**) DMD myoblast line 1 was treated with 5 μg of each TALEN plasmid and dystrophin expression was assessed after 7 days of differentiation by western blot using the NCL-Dys2 antibody. (**d**) DMD myoblast 2 was treated with the indicated amount of each TALEN plasmid and dystrophin expression was assessed after 7 days of differentiation by western blot using the MANDYS8 antibody. Protein from wild-type (WT) human myoblasts differentiated in parallel was diluted 1:100 and loaded as a positive control for full-length dystrophin expression (427 kDa) relative to the truncated Δ48-50 product (412 kDa). DMD, Duchenne muscular dystrophy; GAPDH, glyceraldehyde 3-phosphate dehydrogenase; n.d. not detected; NHEJ, non-homologous end joining; TALEN, transcription activator-like effector nuclease.

Efficient *in situ* frame correction in the absence of selection is a powerful use of NHEJ-based gene correction. Accordingly, we investigated the restoration of dystrophin expression in TALEN-treated bulk populations of DMD myoblast lines derived from two different patients containing different deletions of exons 48-50 in the dystrophin gene. As expected, the frequency of gene modification increased with the dose of electroporated TN3/8-encoding plasmids, with indels detected in up to 12.7 and 6.8% of alleles in the two patient lines as measured by the Surveyor assay (**Figure 4a**,**b**). Following 7 days of myogenic differentiation induced by serum removal, restored dystrophin expression was detected in the bulk cell populations at the predicted size (~412 kDa) relative to expression from wild-type cells (427 kDa) (**Figure 4c**,**d**). The increase in dystrophin protein expression with TALEN dose was concomitant with the level of NHEJ events detected by the Surveyor assay.

### Gene restoration in primary DMD dermal fibroblasts

**Figure 5:**
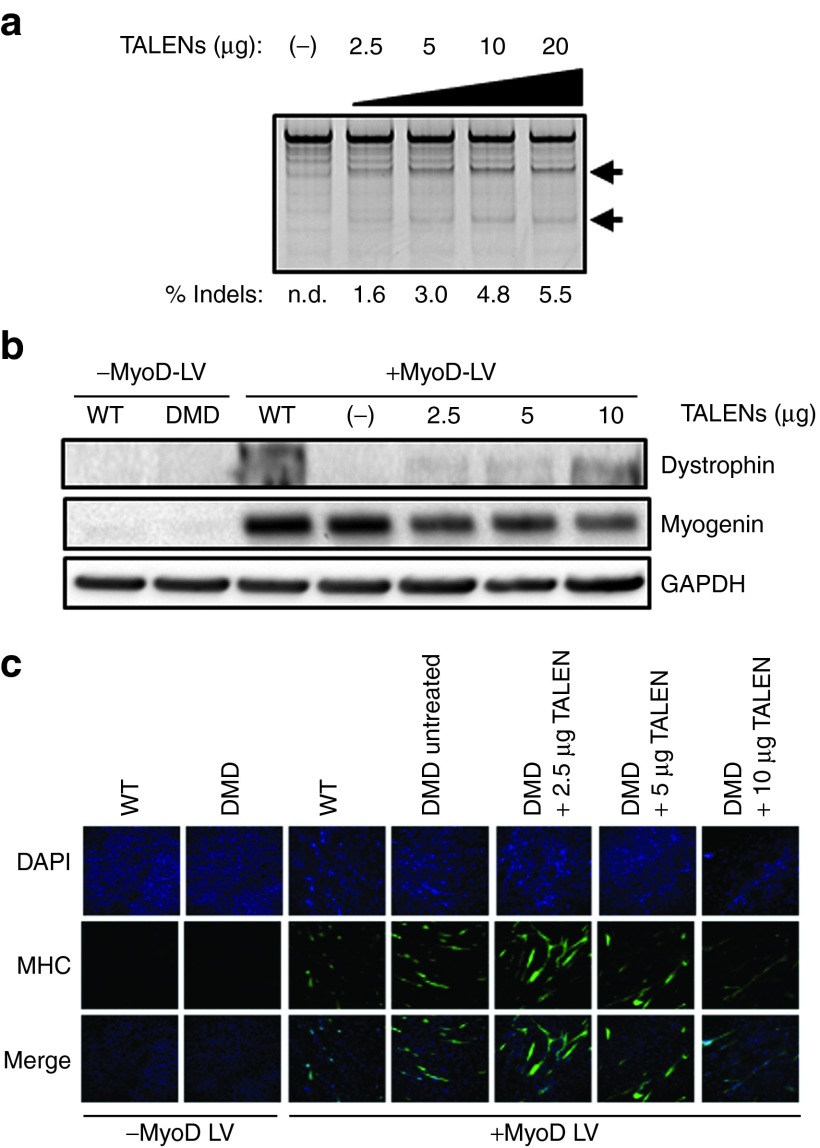
**Dystrophin reading frame restoration in primary dermal fibroblasts**. (**a**) Primary DMD fibroblasts carrying a deletion of exons 46-50 (Δ46-50) were electroporated with increasing doses of the indicated amount of each TALEN plasmid and gene modification rates were quantified with the Surveyor assay. Arrows denote expected cleavage band sizes indicative of NHEJ activity. (**b**) Analysis of myogenin and dystrophin expression (MANDYS8) in wild-type (WT) and DMD fibroblasts after treatment with TN3/8 and 15 days of forced MyoD expression. Protein from WT dermal fibroblasts is included as a positive control for full-length dystrophin expression (427 kDa) relative to the truncated Δ46-50 product (400 kDa). (**c**) Immunofluorescence staining to detect myosin heavy chain (MHC) after MyoD expression by lentiviral gene transfer. DAPI, 4′,6-diamidino-2-phenylindole; DMD, Duchenne muscular dystrophy; GAPDH, glyceraldehyde 3-phosphate dehydrogenase; n.d. not detected; NHEJ, non-homologous end joining; TALEN, transcription activator-like effector nuclease.

The simplicity of this NHEJ-based approach can enable efficient correction in proliferation-limited primary cell lines that may not be amenable to homologous recombination or selection-based gene correction. For example, DMD patient-derived primary dermal fibroblasts carrying a frame-disrupting deletion of exons 46-50 (Δ46-50) were electroporated with plasmids encoding TN3/8, resulting in high frequency gene modification in a dose-dependent manner (**Figure 5a**). These treated fibroblasts were then transduced with a lentivirus expressing MyoD under an inducible promoter to stimulate transdifferentiation into the myogenic lineage and dystrophin expression.^30,31^ Expression of myogenin (**Figure 5b**) and myosin heavy chain (**Figure 5c**) confirmed efficient transdifferentiation of wild-type and DMD patient fibroblasts. Rescued dystrophin expression was detected in TALEN-treated MyoD-induced fibroblasts in a dose-dependent manner at the predicted size of ~400 kDa (**Figure 5b**), similar to the results obtained in skeletal myoblasts (**Figure 4c**,**d**).

### Analysis of off-target effects induced by TN3/8

An important concern for all genome-editing strategies is the potential for off-target gene modification events. TN3/8 does not show significant cytotoxicity and is well tolerated by human cells (**Figure 2b**,**e**,**f**), suggesting specific gene targeting. Potential off-target sites were assessed *in silico* using the TALE-NT 2.0 Paired Target Finder Prediction webserver^32^ to scan the human genome for sequences containing up to four mismatches per TALEN half-site (up to eight total mismatches per target site) separated by spacers of any length between 12 and 23 bases. Importantly, this analysis did not produce any potential off-target sites that met these criteria. To further examine unpredicted off-target DNA modifications, we sequenced the whole exomes of clonally derived DMD myoblasts that we had previously confirmed to contain NHEJ events at the on-target exon 51 locus (**Figure 3c**,**d**). Notably, the only insertion or deletion events characteristic of NHEJ were detected at the on-target exon 51 locus of the dystrophin gene in all four clonal lines analyzed, confirming the specificity of these TALENs (**Table 1**, **Supplementary Table S1**). Consistent with known genomic mutation rates that normally occur during clonal expansion, the exome sequencing revealed several single-nucleotide variants (SNVs) in each clone relative to the parental cell line. Using the TALE-NT 2.0 Paired Target Site Prediction webserver,^32^ the immediate region around each mutation was scanned for any sequence similarity to the TN3/8 target site to determine whether the TALENs could be responsible for the observed SNVs. No target sites with similarity to our TALEN target site with spacers of 1–30 bases were found in the flanking 100 bp of any SNV. Because NHEJ-mediated mutagenesis rarely results in substitutions relative to indels, the detected SNVs are likely to have arisen during clonal expansion as observed in other studies.^12,17^ In summary, there was no apparent off-target activity related to TALEN-mediated, NHEJ-based genetic correction in these clonally derived cells.

## Discussion

NHEJ-exclusive gene correction offers several potential advantages over the HDR pathway. For example, NHEJ does not require a donor template, which may cause nonspecific insertional mutagenesis. In contrast to HDR, NHEJ operates efficiently in all stages of the cell cycle and therefore can be effectively exploited in both cycling and postmitotic cells, such as muscle fibers. This provides a robust, permanent gene restoration alternative to oligonucleotide-based exon skipping^19^ or pharmacologic-forced read-through of stop codons^33^ and could theoretically require as few as one drug treatment. NHEJ-based gene correction using TALENs, as well as other engineered nucleases including meganucleases^24^ and zinc finger nucleases,^34^ is also readily combined with other existing *ex vivo* and *in vivo* platforms for cell- and gene-based therapies, in addition to the plasmid electroporation approach described here. For example, delivery of TALENs by mRNA-based gene transfer or as purified cell-permeable proteins^35^ could enable a DNA-free genome-editing approach that would circumvent any possibility of insertional mutagenesis.

Any of these delivery methods could be utilized with a myriad of cell types currently under investigation for cell-based therapies,^36^ including induced pluripotent stem cells,^37,38^ bone marrow-derived progenitors,^39^ skeletal muscle progenitors,^40^ CD133^+^ cells,^41^ mesoangioblasts,^42^ and MyoD-transduced dermal fibroblasts.^31,32^ In addition, advances in immortalization of human myogenic cells may greatly simplify clonal derivation of genetically corrected myogenic cells.^43^ Significantly, we modified cells *ex vivo* and isolated and expanded clonal populations of immortalized DMD myoblasts that contained a genetically corrected dystrophin gene and were free of nuclease-introduced mutations in protein-coding regions of the genome. Alternatively, transient *in vivo* delivery of nucleases by nonviral or nonintegrating viral gene transfer^18,20,44^ or by direct delivery of purified proteins^35^ containing cell-penetrating motifs may enable highly specific correction *in situ* with minimal or no risk of exogenous DNA integration.

Future studies are warranted to investigate the therapeutic efficacy of this approach and similar permanent gene-editing strategies to correct endogenous genes. For example, rescuing dystrophin expression to produce these “Becker-like” proteins theoretically introduces novel epitopes in the restored C-terminus. Therefore, it will be important to consider potential immune responses following permanent genetic correction of the reading frame,^45^ though current exon-skipping clinical studies suggest a minimal immune response to the restored native gene product.^26,27^ Any reduced function of restored, but truncated, protein products is another potential hurdle to this strategy. In the case of DMD, naturally occurring mutations and their consequences are relatively well understood. It is known that in-frame deletions that occur in the exon 45-55 region contained within the rod domain can produce highly functional dystrophin proteins, and many carriers are asymptomatic or display mild symptoms.^19^ Furthermore, >60% of patients can theoretically be treated by targeting exons in this region of the dystrophin gene.^46^ Collectively, these previous studies indicate that the restored dystrophin proteins created by our approach will be highly functional and alleviate disease symptoms when expressed in skeletal muscle tissue.

Genome editing is a powerful approach for creating custom alterations to the genome, as evidenced by the recent entrance of zinc finger nucleases into clinical trials for disruption of the HIV-1 co-receptor CCR5^21^ and disruption of the glucocorticoid receptor in T cells for glioblastoma treatment. This study utilizes NHEJ-based genome editing to restore the reading frame of the dystrophin gene in patient cells, in contrast to the conventional use of NHEJ for gene knockout. Given the numerous high-throughput methods to engineer new TALENs,^9,10,11^ as well as their apparent lack of cytotoxicity,^7,10^ it should be possible to rapidly extrapolate this NHEJ correction method to other regions of the dystrophin gene as well as other diseases that are caused by a loss of protein function introduced by intragenic insertions, deletions, or aberrant stop codons in non-essential regions, including collagen type VII-associated dystrophic epidermolysis bullosa, Fukuyama congenital muscular dystrophy, and limb-girdle muscular dystrophy type 2B. However, the resulting functionality of these proteins following partial gene correction remains to be determined, particularly for gene deletions and associated phenotypes that are not as well-defined as the region targeted in this study. Therefore HDR-based genome editing for complete restoration of gene deletions is also a valuable approach to pursue in parallel. Nevertheless, NHEJ-based gene correction may provide a versatile therapy for DMD when frame restoration is predicted to permanently correct the native gene and restore protein function.

## Materials and Methods

***Cell culture and transfection.*** HEK293T cells were obtained from the American Tissue Collection Center (ATCC, Manassas, VA) through the Duke Cell Culture Facility and were maintained in Dulbecco's modified Eagle's medium (DMEM) supplemented with 10% bovine calf serum and 1% penicillin/streptomycin. Immortalized myoblasts^47^ (one from a wild-type donor, and two Δ48-50 DMD patient-derived lines) were maintained in skeletal muscle media (PromoCell, Heidelberg, Germany) supplemented with 20% bovine calf serum (Sigma, St Louis, MO), 50 μg/ml fetuin, 10 ng/ml human epidermal growth factor (Sigma), 1 ng/ml human basic fibroblast growth factor (Sigma), 10 μg/ml human insulin (Sigma), 1% GlutaMAX (Invitrogen, Carlsbad, CA), and 1% penicillin/streptomycin (Invitrogen). Primary DMD dermal fibroblasts were obtained from the Coriell Cell repository (Camden, NJ) (GM05162A, Δ46-50) and maintained in DMEM supplemented with 10% fetal bovine serum, 1 ng/ml human basic fibroblast growth factor, and 1% penicillin/streptomycin. All cell lines were maintained at 37 °C and 5% CO_2_. HEK293T cells were transfected with Lipofectamine 2000 (Invitrogen) according to the manufacturer's protocol in 24-well plates. Immortalized myoblasts and primary fibroblasts were transfected by electroporation using the Gene PulserXCell (Bio-Rad, Hercules, CA) with phosphate-buffered saline as an electroporation buffer using optimized conditions for each line (**Supplementary Figure S5**). Transfection efficiencies were measured by delivering an enhanced green fluorescent protein (GFP) expression plasmid and using flow cytometry. These efficiencies were routinely ≥95% for HEK293T and ≥70% for the primary fibroblasts and immortalized myoblasts. For all experiments, the indicated mass of electroporated plasmid corresponds to the amount used for each TALEN monomer.

***TALE nuclease assembly and off-target site prediction.*** TALENs targeted to exon 51 of the human dystrophin gene were designed *in silico* using the TALE-NT webserver.^9^ TALEN target sites were chosen to include half-site targets ~15–19 bp in length, preceded by a 5′-T.^6^ Plasmids encoding these TALENs were assembled using the Golden Gate assembly method^9^ and standard cloning techniques into a modified pcDNA3.1 (Invitrogen) destination vector containing the Δ152/+63 TALEN architecture^6^ derived from the pTAL3 expression vector provided in the Golden Gate kit from Addgene (Cambridge, MA). The *Fok*I endonuclease domains were codon optimized and contained the ELD/KKR obligate heterodimer^48^ and *Sharkey* mutations^49^ as described previously.^50^ Complete TN3/8 sequences are provided in **Supplementary Figure S3**. Potential off-target sites for TALEN pair TN3/8 in the human genome were predicted *in silico* using the Paired Target Finder tool on the TALE-NT 2.0 webserver.^32^ All predicted off-target sites were scanned using the following parameters: recommended score cut-off (3.0), spacers of range 12–23 bp, and upstream base set to “T only”. Valid likely potential off-target sites were only considered as those with up to four mismatches per TALEN half-site–binding sequence (maximum of eight mismatches per TALEN pair target site).

***Cel-I quantification of endogenous gene modification.*** TALEN-induced lesions at the endogenous target site were quantified using the Surveyor nuclease assay, which can detect mutations characteristic of nuclease-mediated NHEJ. After electroporation, cells were incubated for 3 or 10 days at 37 °C and genomic DNA was extracted using the DNeasy Blood and Tissue kit (QIAGEN, Valencia, CA). The target locus was amplified by 30 cycles of PCR with the AccuPrime High Fidelity PCR kit (Invitrogen) using primers 5′-GAGTTTGGCTCAAATTGTTACTCTT-3′ and 5′-GGGAAATGGTCTAGGAGAGTAAAGT-3′. The resulting PCR products were randomly melted and reannealed in a PCR machine with the program: 95 °C for 240 seconds, followed by 85 °C for 60 seconds, 75 °C for 60 seconds, 65 °C for 60 seconds, 55 °C for 60 seconds, 45 °C for 60 seconds, 35 °C for 60 seconds, and 25 °C for 60 seconds with a −0.3 °C/second rate between steps. Following reannealing, 8 μl of PCR product was mixed with 1 μl of Surveyor Nuclease S and 1 μl of Enhancer S (Transgenomic, Omaha, NE) and incubated at 42 °C for 1 hour. After incubation, 6 μl of digestion product was loaded onto a 10% TBE polyacrylamide gel and run at 200 V for 30 minutes. The gels were stained with ethidium bromide and quantified using ImageLab (Bio-Rad) by densitometry as previously described.^50^

***Cytotoxicity assay.*** To quantitatively assess potential TALEN cytotoxicity, HEK293T cells were transfected with 10 ng of a GFP reporter and 100 ng of each nuclease using Lipofectamine 2000 according to the manufacturer's instructions (Invitrogen). The percentage of GFP-positive cells was assessed at 2 and 5 days by flow cytometry. The survival rate was calculated as the decrease in GFP-positive cells from days 2–5 and normalized to cells transfected with an empty nuclease expression vector as described.^28^

***Clone isolation procedure.*** Immortalized DMD myoblasts were electroporated with 10 μg of each TALEN plasmid (20 μg total). After 7 days, isogenic clones were isolated by clonal dilution in hypoxic conditions (5% O_2_) to accelerate myoblast growth. Genomic DNA was extracted from clones using the QuickExtract Kit (Epicentre, Madison, WI) and the target locus amplified by PCR using the Cel-I primers and conditions above. The resulting PCR products were either mixed with equal amounts of PCR product from untreated cells and analyzed by the Surveyor assay described above (**Figure 3a**,**b**) or directly submitted for conventional Sanger sequencing (**Figure 3c**) to identify modified clones.

***Viral transduction and forced MyoD overexpression in primary fibroblasts.*** A total of 300,000 fibroblasts were plated and transduced in 10 cm plates with a lentiviral vector encoding a full-length human MyoD cDNA under the control of a dox-inducible promoter and a constitutive puromycin resistance cassette. Two days post-transduction, fibroblasts were selected for 6 days in 1 μg/ml puromycin (Sigma) to enrich for transduced cells. Fibroblasts were then plated at a density of 200,000 cells in 10 cm dishes and MyoD expression was induced by adding 3 μg/ml doxycycline (Fisher Scientific, Waltham, MA) to the media, which was exchanged every 2 days.

***Western blot analysis.*** To assess dystrophin expression, immortalized myoblasts were differentiated into myofibers by replacing the growth medium with DMEM supplemented with 1% insulin-transferrin-selenium (Invitrogen) and 1% antibiotic/antimycotic (Invitrogen) for 4–7 days. Fibroblasts were transdifferentiated into myoblasts by inducing MyoD overexpression and incubating the cells in DMEM supplemented with 1% insulin-transferrin-selenium (Invitrogen), 1% antibiotic/antimycotic (Invitrogen), and 3 μg/ml doxycycline for 15 days. TALEN expression was assessed at 3 days after transfecting HEK293T cells. Cells were collected and lysed in RIPA buffer (Sigma) supplemented with a protease inhibitor cocktail (Sigma) and the total protein amount was quantified using the bicinchoninic acid assay according to the manufacturer's instructions (Pierce, Rockford, IL). Samples were then mixed with NuPAGE loading buffer (Invitrogen) and 5% β-mercaptoethanol and heated to 85 °C for 10 minutes. Twenty-five microgram of protein were separated on 4–12% NuPAGEBis-Tris gels (Invitrogen) with MES buffer (Invitrogen). Proteins were transferred to nitrocellulose membranes for 1–2 hours in transfer buffer containing 10–20% methanol and 0.01% sodium dodecyl sulfate. The blot was then blocked for 1 hour with 5% milk-Tris-buffered saline and Tween 20 at room temperature. Blots were probed with the following primary antibodies: NCL-Dys2 (1:25; Leica, Buffalo Grove, IL), MANDYS8 (1:100; Sigma), GAPDH (1:5,000; Cell Signaling, Danvers, MA), anti-FLAG-HRP (1:2,000; Cell Signaling), or anti-myogenin F5D (1:200; Santa Cruz Biotechnology, Santa Cruz, CA). Dystrophin expression was detected using MANDYS8 in DMD myoblast line 2 and the DMD fibroblast line or NCL-Dys2 in DMD myoblast line 1. TALEN expression was detected using anti-FLAG. Blots were then incubated with mouse or rabbit horseradish peroxidase-conjugated secondary antibodies (Santa Cruz Biotechnology) and visualized using the ChemiDoc chemilumescent system (Bio-Rad) and Western-C ECL substrate (Bio-Rad).

***Immunofluorescence.*** Fibroblasts were plated on cover slips in 24-well plates at a density of 30,000 cells/well and MyoD expression was induced for 15 days as described above. Cells were then fixed in 4% paraformaldehyde and blocked for 1 hour at room temperature with phosphate-buffered saline containing 5% bovine serum albumin, 2% goat serum, and 0.2% Triton X-100. Cells were then stained overnight at 4 °C with MF20 (1:200; Developmental Studies Hybridoma Bank, Iowa City, IA) primary antibody and then for 1 hour at room temperature with anti-mouse AlexaFluor 488 (Molecular Probes, Eugene, OR) secondary antibody. Cover slips were mounted with ProLong Gold antifade (Molecular Probes).

***Exome sequencing and analysis.*** We analyzed the exomes of four clonally derived DMD myoblast lines carrying known TALEN-mediated deletions in exon 51 of the dystrophin gene, as well as the parent line for these cells. Genomic DNA was isolated using the DNeasy Blood and Tissue Kit (QIAGEN) and 3 μg of DNA were submitted to the Duke Institute for Genome Sciences and Policy's Genome Sequencing & Analysis Core. Illumina-compatible libraries were made and enriched for exonic regions using the SureSelect Human All Exon V4 Kit (Agilent, Santa Clara, CA). Five total libraries were prepared from the four treatment samples and one parental line reference sample. The libraries were indexed and sequenced on one lane of Illumina HiSeq2000 (100-bp paired-end sequencing). Bioinformatics analyses were performed by Duke Genome Sequencing & Analysis Core. The analysis pipeline includes the initial QC to remove sequencing adaptors and low quality bases to facilitate mapping. Sequence depth of targeted regions was calculated as >97% at 10x coverage, >91% at 20x coverage, and >82% for 30x coverage (**Supplementary Table S1**). Each sequencing reaction generated >64 million reads with >93% of reads above a quality score of 30 and an overall mean quality score of >36.4. High quality reads were mapped to the human reference genome (hg19) using bwa 0.5.9. An exome capture pipeline developed at the Duke Sequencing Core was used to assess the exome capture efficiency. Picard v1.74 is used for removing PCR duplicates. The GATK (v1.6-13) toolkit (Broad Institute, Cambridge, MA) is used for variant calling, read realignment around INDELs, quality score recalibration, and QC filtering. The filtering step discards the variants with (i) low coverage (<30x), (ii) strand-bias, (iii) low SNP quality score (<50), and (iv) low allelic frequency (<0.5). Each candidate point mutation or INDEL were reviewed manually by Integrated Genomics Viewer to identify false negative artifacts due to insufficient coverage of the parental line. Identical point mutations and INDELs that occurred in more than two of the four clones were verified as artifacts due to coverage of the reference parent cell line and were discarded. Common point mutations and INDELs were removed by comparing to human dbSNP135. The remaining point mutations and INDELs were annotated using Annovar (25 May, 2012 version) and classified using a perl script written by the Duke Sequencing Core. The non-exonic point mutations were not considered. All point mutations and INDELs were individually visualized and validated on IGV. The flanking 100 bp of each validated mutation was screened for any potential sequence similarity to the TN3/8 target site using the Paired Target Finder tool on the TALE-NT 2.0 webserver^32^ using the parameters: recommended score cut-off (3.0), spacers of range 1–30 bp, and upstream base set to “T only”.

**SUPPLEMENTARY MATERIAL** **Figure S1.** Target sequences and RVDs for TALENs in this study. **Figure S2.** Optimization of cytotoxicity assay using Lipofectamine 2000 in 293T cells. **Figure S3.** Complete amino acid sequences of TALENs TN3 and TN8 used in this study. **Figure S4.** Chromatograms of clones from **Figure 3.** **Figure S5.** Optimization of electroporation conditions for myoblasts. **Table S1.** Exome capture statistics.

## Figures and Tables

**Table 1:**
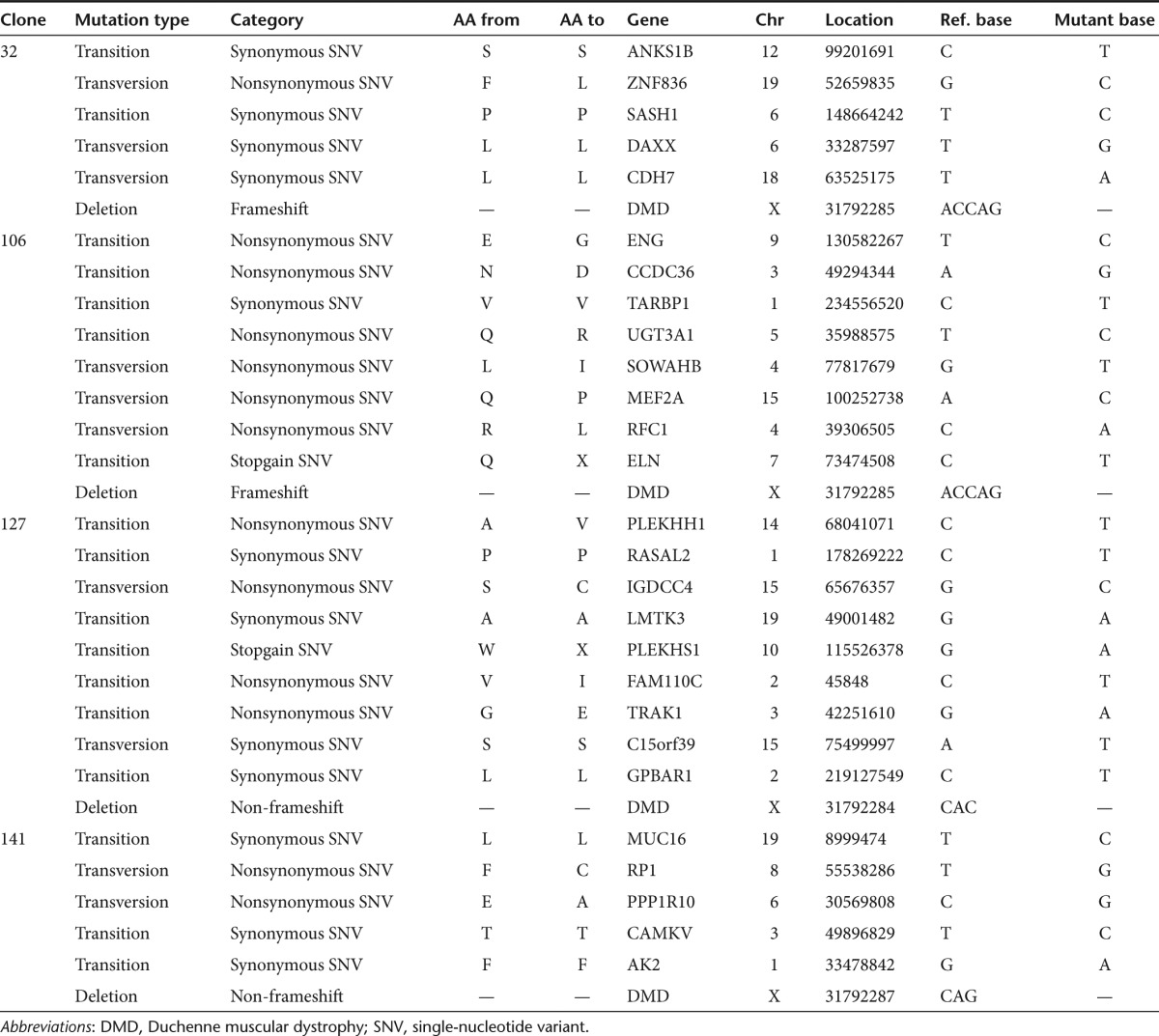
Summary of clonal sequence variants detected by exome sequencing
