# Supplementary material for: Reading Frame Correction by Targeted Genome Editing Restores Dystrophin Expression in Cells From Duchenne Muscular Dystrophy Patients
Source: Mol Ther. 2013 Jun 4;21(9):1718–26. doi: 10.1038/mt.2013.111 (PMC3776627; doi:10.1038/mt.2013.111)
Supplement: Supplementary Information [file mt2013111x1.pdf]

Reading Frame Correction by Targeted Genome Editing Restores Dystrophin Expression in Cells from Duchenne Muscular Dystrophy Patients

David G. Ousterout,[1] Pablo Perez-Pinera,[1] Pratiksha I. Thakore,[1] Ami M. Kabadi,[1] Matthew T. Brown,[1], Xiaoxia Qin[2], Olivier Fedrigo[2], Vincent Mouly,[4] Jacques P. Tremblay,[5] Charles A. Gersbach[1-3]

[1] Department of Biomedical Engineering, Duke University, Durham, North Carolina, United States of America, 27708

[2]Institute for Genome Sciences and Policy, Duke University, Durham, North Carolina, United States of America, 27708

[3]Department of Orthopaedic Surgery, Duke University Medical Center, Durham, North Carolina, United States of America, 27710

[4]Thérapie des maladies du muscle strié/Institut de Myologie UM76, Université Pierre et Marie Curie, INSERM-U974; CNRS-UMR7215, Paris, France

[5]Axe Neuroscience, Centre de Recherche de CHU de Québec and Département de Médecine Moléculaire, Faculté de Médecine, Université Laval, Québec, Québec, Canada

[*]Address for correspondence:
Charles A. Gersbach, Ph.D.
Department of Biomedical Engineering
Room 136 Hudson Hall, Box 90281
Duke University
Durham, NC 27708-0281
Phone: 919-613-2147
Fax: 919-668-0795
Email: charles.gersbach@duke.edu

	Target sequence (5'-3')	RVDs
**TN1**	attttagctcctact	NI NG NG NG NG NI NN HD NG HD HD NG NI HD NG
**TN2**	tttagctcctactcaga	NG NG NG NI NN HD NG HD HD NG NI HD NG HD NI NN NI
**TN3**	agctcctactcagact	NI NN HD NG HD HD NG NI HD NG HD NI NN NI HD NG
**TN4**	cctactcagactgtt	HD HD NG NI HD NG HD NI NN NI HD NG NN NG NG
**TN5**	aaccacaggttgtgtca	NI NI HD HD NI HD NI NN NN NG NG NN NG NN NG HD NI
**TN6**	agtaaccacaggttgt	NI NN NG NI NI HD HD NI HD NI NN NN NG NG NN NG
**TN8**	ccttagtaaccacaggt	HD HD NG NG NI NN NG NI NI HD HD NI HD NI NN NN NG

Supplementary Figure 1: Target sequences and RVDs for TALENs in this study. All target sequences are preceded by a prerequisite 5'-T.

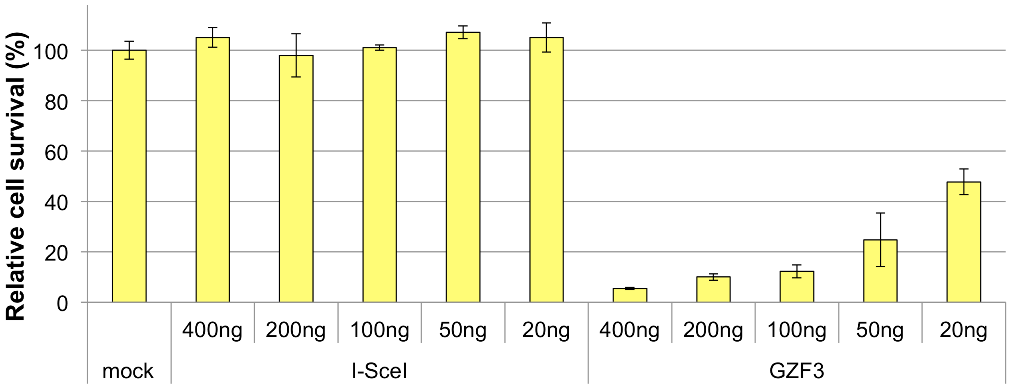

Supplementary Figure 2: Optimization of cytotoxicity assay using Lipofectamine 2000 in 293T cells. Varying amounts of plasmid encoding the non-toxic endonuclease I-SceI and toxic zinc-finger nuclease GZF3 were transfected into 293T cells and assessed for relative survival rates post-transfection. Based on these data, 100 ng of nuclease expression plasmid was used for the cytotoxicity studies.

TN3 (Fok-ELDS):

MDYKDHDGDYKDHDIDYKDDDDKMAPKKKRKVGRGSVDLRTLGYSQQQQEKIKPKVRST
VAQHHEALVGHGFTHAHIVALSQHPAALGTVAVTYQHIITALPEATHEDIVGVGKQWSG
ARALEALLTDAGELRGPPLQLDTGQLVKIAKRGGVTAMEAVHASRNALTGAPLNLTPDQ
VVAIASNIGGKQALETVQRLLPVLCQDHGLTPDQVVAIASNNGGKQALETVQRLLPVLC
QDHGLTPDQVVAIASHDGGKQALETVQRLLPVLCQDHGLTPDQVVAIASNGGGKQALET
VQRLLPVLCQDHGLTPDQVVAIASHDGGKQALETVQRLLPVLCQDHGLTPDQVVAIASH
DGGKQALETVQRLLPVLCQDHGLTPDQVVAIASNGGGKQALETVQRLLPVLCQDHGLTP
DQVVAIASNIGGKQALETVQRLLPVLCQDHGLTPDQVVAIASHDGGKQALETVQRLLPV
LCQDHGLTPDQVVAIASNGGGKQALETVQRLLPVLCQDHGLTPDQVVAIASHDGGKQAL
ETVQRLLPVLCQDHGLTPDQVVAIASNIGGKQALETVQRLLPVLCQDHGLTPDQVVAIA
SNNGGKQALETVQRLLPVLCQDHGLTPDQVVAIASNIGGKQALETVQRLLPVLCQDHGL
TPDQVVAIASHDGGKQALETVQRLLPVLCQDHGLTPDQVVAIASNGGGKQALESIVAQL
SRPDPALAALTNDHLVALACLGGRPAMDAVKKGLPHAPELIRRVNRRIGERTSHRVAQL
VKSELEEKKSELRHKLKYVPHEYIELIEIARNPTQDRILEMKVMEFFMKVYGYRGEHLG
GSRKPDGAIYTVGSPIDYGVIVDTKAYSGGYNLPIGQADEMERYVEENQTRDKHLNPNE
WWKVYPSSVTEFKFLFVSGHFKGNYKAQLTRLNHITNCNGAVLSVEELLIGGEMIKAGT
LTLEEVRRKFNNGEINF

TN8 (Fok-KKRS):

MDYKDHDGDYKDHDIDYKDDDDKMAPKKKRKVGRGSVDLRTLGYSQQQQEKIKPKVRST
VAQHHEALVGHGFTHAHIVALSQHPAALGTVAVTYQHIITALPEATHEDIVGVGKQWSG
ARALEALLTDAGELRGPPLQLDTGQLVKIAKRGGVTAMEAVHASRNALTGAPLNLTPDQ
VVAIASHDGGKQALETVQRLLPVLCQDHGLTPDQVVAIASHDGGKQALETVQRLLPVLC
QDHGLTPDQVVAIASNGGGKQALETVQRLLPVLCQDHGLTPDQVVAIASNGGGKQALET
VQRLLPVLCQDHGLTPDQVVAIASNIGGKQALETVQRLLPVLCQDHGLTPDQVVAIASN
NGGKQALETVQRLLPVLCQDHGLTPDQVVAIASNGGGKQALETVQRLLPVLCQDHGLTP
DQVVAIASNIGGKQALETVQRLLPVLCQDHGLTPDQVVAIASNIGGKQALETVQRLLPV
LCQDHGLTPDQVVAIASHDGGKQALETVQRLLPVLCQDHGLTPDQVVAIASHDGGKQAL
ETVQRLLPVLCQDHGLTPDQVVAIASNIGGKQALETVQRLLPVLCQDHGLTPDQVVAIA
SHDGGKQALETVQRLLPVLCQDHGLTPDQVVAIASNIGGKQALETVQRLLPVLCQDHGL
TPDQVVAIASNNGGKQALETVQRLLPVLCQDHGLTPDQVVAIASNNGGKQALETVQRLL
PVLCQDHGLTPDQVVAIASNGGGKQALESIVAQLSRPDPALAALTNDHLVALACLGGRP
AMDAVKKGLPHAPELIRRVNRRIGERTSHRVAQLVKSELEEKKSELRHKLKYVPHEYIE
LIEIARNPTQDRILEMKVMEFFMKVYGYRGEHLGGSRKPDGAIYTVGSPIDYGVIVDTK
AYSGGYNLPIGQADEMQRYVKENQTRNKHINPNEWWKVYPSSVTEFKFLFVSGHFKGNY
KAQLTRLNRKTNCNGAVLSVEELLIGGEMIKAGTLTLEEVRRKFNNGEINF

Supplementary Figure 3: Complete amino acid sequences of TALENs TN3 and TN8 used in this study.

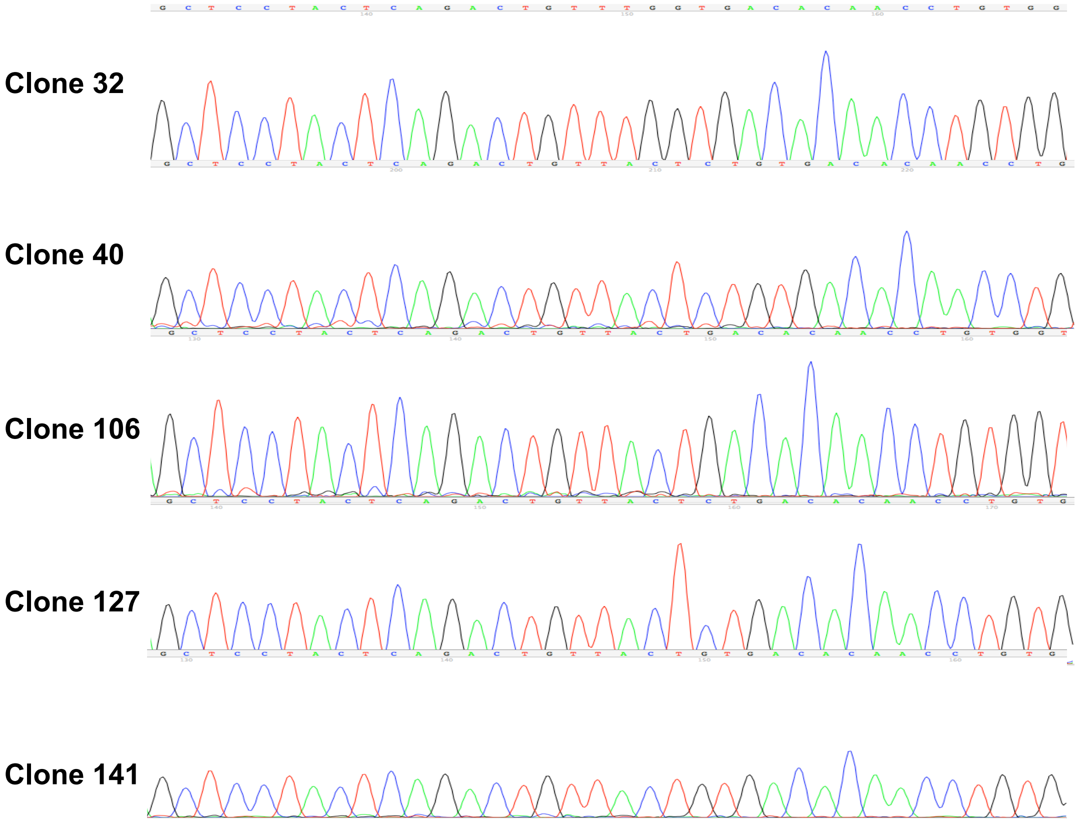

Supplementary Figure 4: Chromatograms of clones from Figure 3.

a

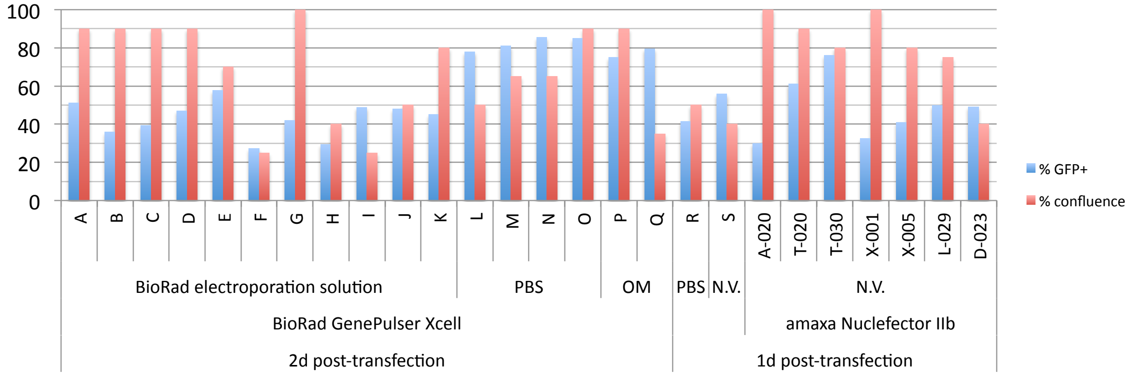

b

Program	Conditions
A	1000uF, 120V, 1M cells/200uL, 10ug GFP + 10ug empty vector
B	500uF, 120V, 1M cells/200uL, 10ug GFP + 10ug empty vector
C	300uF, 120V, 1M cells/200uL, 10ug GFP + 10ug empty vector
D	500uF, 100V, 1M cells/200uL, 10ug GFP + 10ug empty vector
E	500uF, 150V, 1M cells/200uL, 10ug GFP + 10ug empty vector
F	500uF, 200V, 1M cells/200uL, 10ug GFP + 10ug empty vector
G	1000uF, 100V, 1M cells/200uL, 10ug GFP + 10ug empty vector
H	1000uF, 150V, 1M cells/200uL, 10ug GFP + 10ug empty vector
I	1000uF, 100V, 0.2M cells/200uL, 10ug GFP + 10ug empty vector
J	1000uF, 100V, 0.4M cells/200uL, 10ug GFP + 10ug empty vector
K	1000uF, 100V, 1M cells/200uL, 10ug GFP + 10ug empty vector
L	950uF, 190V, 0.5M cells/150uL, 5ug GFP
M	950uF, 190V, 1M cells/200uL, 10ug GFP
N	950uF, 160V, 0.5M cells/150uL, 5ug GFP
O	950uF, 160V, 1M cells/200uL, 10ug GFP
P	950uF, 220V, 2M cells/300uL, 10ug GFP
Q	950uF, 100V, 1M cells/200uL, 10ug GFP
R	950uF, 190V, 1M cells/200ul, 2ug pmaxGFP
S	950uF, 190V, 1M cells/100uL, 2ug pmaxGFP

Supplementary Figure 5: Optimization of electroporation conditions for myoblasts. (a) DMD myoblast cells (cell line 1) were electroporated using BioRad Gene Pulser Xcell or Amaxa Nucleofector IIb devices using the indicated programs. Several different buffers were tested, including BioRad electroporation solution, Sigma phosphate-buffered saline product #D8537 (PBS), Invitrogen OptiMEM I (OM), or Amaxa Nucleofector solution V (N.V.). Conditions using the GenePulser device used infinite resistance. For nucleofection, 1 million cells/100 μL nucleofection solution and 2 μg of GFP vector were used according to the manufacturer's specifications. Electroporation using the GenePulser device with program O in PBS solution was selected as the optimal conditions for electroporating myoblasts. (b) Conditions used to optimize BioRad Gene Pulser Xcell electroporation.

Sample Name	D0106	DO127	DO141	DO32	DOWT	Agilent-Human All Exon V4
capture efficiency						
Reads onTarget	79.33	79.28	79.27	75.95	79.35	75
Reads On-Target+/-100bp	86.84	88.96	89.15	86.11	89.15	85
Coverage						
1x	99.87	99.88	99.88	99.87	99.88	99
10x	97.4	97.71	97.48	97.53	97.46	90
20x	91.26	92.28	91.41	91.62	91.41	80
30x	82.66	84.45	82.8	83.27	82.94	
50x	63.51	66.35	63.54	64.35	63.9	
100x	28.13	31.12	27.85	28.45	28.62	

Supplementary Table 1: Exome capture statistics. DOWT is the parent DMD myoblast cell line used as the reference sample for analysis. DO32, DO106, DO127, and DO141 are the four clonally derived DMD myoblast lines carrying predetermined on-target NHEJ events at the exon 51 dystrophin locus.
